# Influence of Cold Plasma Treatment on Cellulose Modification with Different Oxidizing Agents

**DOI:** 10.3390/ma18051066

**Published:** 2025-02-27

**Authors:** Denis Mihaela Panaitescu, Sorin Ionuţ Vizireanu, Gabriela Mădălina Oprică, Cătălina Diana Uşurelu, Cristian Stancu, Veronica Sătulu, Marius Ghiurea, Cristian-Andi Nicolae, Monica Florentina Raduly, Adriana Nicoleta Frone

**Affiliations:** 1 National Institute for Research & Development in Chemistry and Petrochemistry—ICECHIM, 202 Splaiul Independentei, 060021 Bucharest, Romania; madalina.oprica@icechim.ro (G.M.O.); cristian.nicolae@icechim.ro (C.-A.N.); monica.raduly@icechim.ro (M.F.R.); adriana.frone@icechim.ro (A.N.F.); 2 Low Temperature Plasma Laboratory, National Institute for Laser, Plasma and Radiation Physics, 077125 Măgurele, Romania; cristian.stancu@infim.ro (C.S.); veronica.satulu@infim.ro (V.S.); 3 Faculty of Chemical Engineering and Biotechnology, National University of Science and Technology Politehnica Bucharest, 011061 Bucharest, Romania

**Keywords:** microcrystalline cellulose, atmospheric cold plasma, oxidizing agents, defibrillation, nanofibers

## Abstract

Cellulose is a versatile biopolymer increasingly applied in medicine and industry due to its biodegradability and biocompatibility, along with the renewability and large availability of source materials. However, finding simple, eco-friendly, and effective methods to modify cellulose to provide it with new functionalities remains a challenge. This work presents a new, inexpensive, and eco-friendly method to chemically modify microcrystalline cellulose (MCC) by the submerged cold plasma treatment of an aqueous suspension of MCC containing different oxidizing agents, such as hydrogen peroxide (H_2_O_2_), sodium hypochlorite (NaClO), or sodium periodate (NaIO_4_). Fourier-transform Infrared (FTIR) and X-ray photoelectron spectroscopy (XPS) showed that plasma treatment intensified the oxidizing effect of H_2_O_2_, NaClO, and NaIO_4_, with plasma-assisted NaClO treatment yielding the highest MCC oxidation level. XPS indicated that the plasma-assisted oxidations also resulted in different degrees of chemical degradation of MCC, a finding further supported by the thermogravimetric analysis (TGA) results. X-ray diffraction (XRD) data revealed a different effect of the oxidizing agents on the crystalline and amorphous regions in MCC. Scanning electron microscopy (SEM) images showed that the combined treatment with plasma and chemical oxidizing agents led to MCC fragmentation and varying degrees of defibrillation into nanofibers.

## 1. Introduction

Renewable resources are currently considered a viable alternative to petroleum feedstock for the design of new eco-friendly materials that can prevent pollution and global warming [1,2,3]. Cellulose is a versatile biopolymer increasingly applied in medicine, food, pharmaceutics, packaging, electronics, and other applications due to its biodegradability, biocompatibility, good mechanical and optical properties, along with the renewability and large availability of the sources that are used for its production [1,4]. Cellulose micro- or nanofibers can be obtained from a multitude of sources and, especially, from agricultural residues, which is beneficial for the environment by reducing the high pressure of waste disposal [5,6]. Transforming cellulose into high value-added products requires a large number of processes including extraction, purification, and surface functionalization. The abundant OH groups on the surface of cellulose are involved in multiple hydrogen bonds with a stabilizing effect [7], but also provide a basis for further chemical modifications. Several surface modification routes have been proposed for enhancing the interfacial adhesion of cellulose to various polymers in composite materials, or for improving its barrier, water repealing, or absorption properties [8,9,10]. To achieve these improvements, cellulose has been surface modified by esterification, etherification, oxidation, grafting, and crosslinking reactions [11,12].

**Scheme 1:**
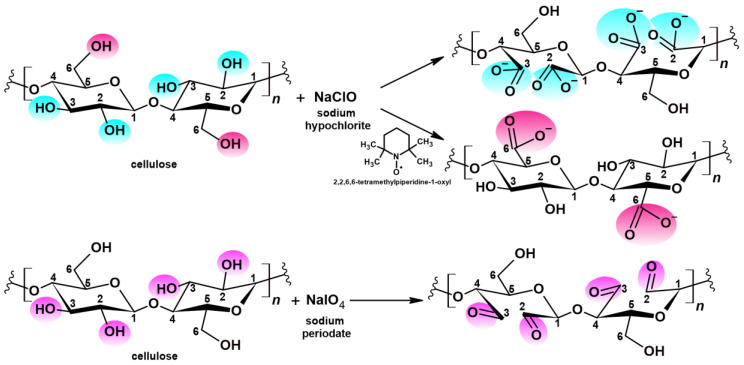
Chemo-selective oxidation of cellulose in the presence of different oxidizing systems.

The oxidation of the OH groups of cellulose is a common route to obtain more reactive cellulose-based materials that can be used as such in various applications or can serve as a basis for further functionalization of cellulose [13,14,15,16]. Hydrogen peroxide, chlorine dioxide, nitrogen dioxide, ammonium persulfate, sodium hypochlorite, and sodium periodate are among the most used cellulose oxidizing agents [15,17]. Cellulose oxidation may also take place during the bleaching treatment of lignocellulosic sources for the removal of hemicelluloses and lignin, under the action of the chlorine-based compounds or hydrogen peroxide commonly used for this purpose [18,19,20,21,22]. When used as a bleaching and/or oxidizing agent, hydrogen peroxide leads to unselective oxidation of cellulose [23], unless special conditions or catalysts are employed [24]. Similarly, sodium hypochlorite can be used to isolate cellulose from different sources by a one-step process, which probably proceeds with the oxidation of the OH groups from the C2 and C3 atoms in cellulose to carboxyl groups [19,25] or to selectively oxidize the primary hydroxyl groups (C6-OH) of cellulose in the presence of nitroxyl radicals such as TEMPO (2,2,6,6-tetramethylpiperidine-1-oxyl) [26]. Contrarily, sodium periodate is a highly chemo-selective oxidizing agent, leading to the cleavage of the C2(OH)-C3(OH) bonds in cellulose and formation of dialdehydes [16,20,27]. The possible chemical reactions that can take place during cellulose oxidation as previously specified are presented in Scheme 1.

Most of the routes known so far to obtain oxidized cellulose and nanocellulose have shortcomings related to low effectiveness, severe reaction conditions, and cellulose depolymerization, along with the expensiveness or toxicity of some compounds and reaction products [1,12,16,22]. In general, the reactions for the isolation (and modification) of (nano)cellulose are intensive time- and energy-consuming processes, often requiring high pressure and temperature conditions [28,29]. Several methods have been experimented to reduce the consumption of energy and to make the processes for (nano)cellulose isolation and modification “greener”, by cumulating the isolation and modification of nanocellulose in a single step reaction [19,25] or by applying only green hydrogen peroxide extraction and/or modification of cellulose [18,24]. In addition, more environmentally friendly pretreatments consisting of ultrasonication, electron beam irradiation, or plasma treatments were applied instead of the expensive and energy-intensive conventional processes [30].

Cold plasma is an environmentally friendly, cost-effective process that ensures a rapid modification of surfaces in safe conditions [4,31,32]. The application of the atmospheric plasma method emerged as a clean and effective route to initiate or enhance the reactions on the surface of cellulose [33]. It has been reported that plasma technology can be applied on a microcrystalline cellulose (MCC) or filter paper substrate prior to sulfuric acid hydrolysis or it can be applied directly to these cellulose materials suspended in sulfuric acid to produce nanocellulose by combined oxidation–hydrolysis processes [34]. However, more environmentally friendly processes were also coupled with plasma treatment to obtain or modify nanocellulose [2,29]. For example, a combined cold plasma/Fenton pretreatment of ground corn stalks led to the breaking of the lignocellulose structure and removal of a great part of lignin, favoring the subsequent enzymatic hydrolysis [29]. The simple treatment of a cellulose powder by atmospheric dielectric barrier discharge (DBD) plasma induced the oxidation of cellulose by the cleavage of the hydrogen bonds and β-1,4-glucosidic bonds followed by the destruction of the pyranose ring through the cleavage of the ether bond [33]. Similar results were reported for cellulose extracted from sugarcane bagasse through a sequence of treatments with nitric acid, sodium hydroxide, and hydrogen peroxide, which was further suspended in water and plasma-treated in a DBD reactor [4].

In our previous work, cellulose nanofibers 40–60 nm in width were obtained from MCC through an eco-friendly technology consisting of cold plasma treatment combined with ultrasonication [31]. A particular innovation brought by this method was the application of a plasma torch source previously ignited in open air directly in the MCC water suspension [31]. A comparison of a TEMPO oxidation reaction and a plasma treatment, which were applied to microcrystalline cellulose, showed that both modified celluloses acted as efficient reinforcing agents for a poly(3-hydroxybutyrate) matrix, although the TEMPO oxidation reaction led to a much more effective surface functionalization of cellulose than the plasma treatment [35]. A plasma torch source and a DBD with a filamentary jet were also used as eco-friendly alternatives to chemical treatments for the surface functionalization of a nanocellulose that was further employed in the preparation of polymer nanocomposites [32]. It was observed that the content of carboxyl groups, determined by X-ray photoelectron spectroscopy (XPS), was higher on the surface of nanocellulose treated in Ar plasma than in Ar/O_2_ plasma [32]. This result showed that the maximum O_2_ flow, which can be used in this plasma treatment method, was not enough for a substantial oxidation of cellulose. Therefore, in this work, a DBD plasma source was submerged in the MCC water suspensions containing different oxidizing agents, namely hydrogen peroxide, sodium hypochlorite, and sodium periodate. The surface-modified celluloses treated with cold plasma in the presence of the oxidizing agents or with the oxidizing agents alone were characterized by Fourier transform infrared (FTIR) spectroscopy, XPS, scanning electron microscopy (SEM), atomic force microscopy, X-ray diffraction (XRD), and thermogravimetric analysis (TGA) to demonstrate the effect of cold plasma and oxidizing agents on the cellulose defibrillation and surface oxidation.

To the best of our knowledge, this is the first time plasma was coupled with oxidizing agents to obtain oxidized defibrillated cellulose, which can be used in medicine as a hemostatic agent or antimicrobial and absorbent material and, after further functionalization, in many other applications.

## 2. Materials and Methods

### 2.1. Materials

MCC from cotton linters, with a mean width of 20 µm, was purchased from Sigma Aldrich (USA); hydrogen peroxide (H_2_O_2_, 10%) was obtained from Chimreactiv SRL (Bucharest, Romania); sodium hydroxide (NaOH, 98%) was acquired from Merck (Darmstadt, Germany); sodium periodate (NaIO_4_, 99%) was purchased from Alfa Aesar (Karlsruhe, Germany); and sodium hypochlorite (NaClO, 9%) was kindly donated by Oltchim Rm-Valcea (Ramnicu Valcea, Romania).

### 2.2. Preparation of the Cellulose Suspensions Containing Various Oxidizing Agents

The reference cellulose suspension (MCC, 1.4 wt%) was obtained by dispersing 1 g MCC in 70 mL bi-distilled water followed by ultrasonication in an ultrasonic bath Elmasonic S40H (Elma, Singen, Germany) with a 37 kHz ultrasonic frequency for 30 min at room temperature. The cellulose suspension containing H_2_O_2_ as an oxidizing agent (MCC-H_2_O_2_) was obtained by dispersing 1 g MCC in 16 mL bi-distilled water, adding 4 mL of 0.1M NaOH solution, ultrasonication for 15 min, followed by the addition of 50 mL H_2_O_2_ (~10%), and ultrasonication again for another 15 min. The anhydroglucose unit (AGU)/H_2_O_2_ molar ratio of 0.04 was chosen as an intermediate value based on the literature [36,37]. The cellulose suspension with NaClO (MCC-NaClO) was obtained by dispersing 1 g MCC in 20 mL bi-distilled water followed by ultrasonication for 15 min, the addition of 50 mL NaClO (9%), and then ultrasonication again for another 15 min. An AGU/NaClO molar ratio of about 0.10 was used in this work, similar to previous reports [19,38]. The cellulose suspension with NaIO_4_ as the oxidizing agent (MCC-NaIO_4_) was prepared using 1 g MCC dispersed in 70 mL bi-distilled water by ultrasonication (15 min), which was mixed with 0.8 g NaIO_4_ and ultrasonicated for another 15 min. In this case, the vessel was covered with aluminum foil to avoid periodate decomposition [39]. According to previous results [39], the AGU/NaIO_4_ molar ratio of 1.65, which was used here, can lead to an aldehyde content of 0.432 mmol/g in 3 h at room temperature. The cellulose suspensions containing the oxidizing agents and the reference suspension (without additives) were divided into two groups: one group was kept at room temperature for 3 h and the other was plasma-treated as described below. Afterwards, the samples were stored in a refrigerator.

### 2.3. Plasma Treatment of the Cellulose Suspensions

A DBD plasma source working at atmospheric pressure was ignited in open air and submerged in the MCC suspensions containing the oxidizing agents for 15 min. The DBD plasma jet was generated in 3000 sccm argon (Ar) flow at 100 W. More details about the DBD plasma source can be found in [40], where a similar source was used for the degradation of methylene blue.

The MCC reference and the suspensions containing the oxidizing agents were denoted as MCC, MCC-H_2_O_2_, MCC-NaClO, and MCC- NaIO_4_, while the plasma-treated suspensions were denoted as MCC-Ar, MCC-H_2_O_2_-Ar, MCC-NaClO-Ar, and MCC-NaIO_4_-Ar. Further, the plasma-treated suspensions and the untreated ones were washed with plenty of distilled water (10/1 *v*/*v*) and the solids were separated by centrifugation using a Universal 320R Ultracentrifuge (Hettich, Kirchlengern, Germany) at 5000 rpm for 5 min. This operation was repeated six times. Parts of these suspensions were kept for at least 24 h in a freezer and lyophilized (−80 °C, 0.008 mbar) for 48 h, while the remaining amounts of cellulose suspensions were stored at 4 °C for further analyses.

### 2.4. Characterization

#### 2.4.1. Surface Chemical Characterization by FTIR and XPS Analyses

FTIR analysis was carried out in ATR (Attenuated Total Reflection) mode on lyophilized cellulose powders using a Jasco FTIR 6300 spectrometer (Jasco Int. Co. Ltd., Tokyo, Japan) equipped with a Specac ATR Golden Gate (Specac Ltd., Orpington, UK) in the 400–4000 cm^−1^ range. A total of 128 scans at a resolution of 4 cm^−1^ were acquired for each spectrum at room temperature.

The ESCALAB™ XI+ spectrometer (Thermo Scientific, Hillsboro, OR, USA) with a monochromatic Al Kα source at 1486.6 eV was used to record the XPS spectra of cellulose samples. For this, a few drops of the cellulose suspensions previously ultrasonicated for 10 min were placed on silicon wafers and dried under vacuum at 30 °C for 24 h. Both survey (step of 1 eV, pass energy of 100 eV) and high-resolution (step of 0.1 eV, pass energy of 20 eV) spectra in the C1s and O1s regions were recorded.

#### 2.4.2. Thermal Characterization

TGA and derivative curves (DTG) of the cellulose samples were obtained with a TA-Q5000 equipment from TA Instruments (New Castle, DE, USA) using nitrogen as a purge gas (40 mL/min). For this, samples weighing 10–14 mg were heated from room temperature to 700 °C at 10 °C/min.

#### 2.4.3. Morphological Investigation by SEM and AFM

The morphological characteristics of the cellulose samples were investigated using a Hitachi TM4000Plus microscope (Hitachi, Tokyo, Japan) in backscattered electron (BSE) mode at an accelerating voltage of 15 kV. Prior to the SEM analysis, the lyophilized samples were loaded on pin stubs using adhesive carbon tapes and sputter-coated with gold (5 nm) using a Q150R Plus (Quorum Technologies, Lewes, UK).

The morphological aspects of plasma-treated oxidized celluloses were also evaluated using a MultiMode 8 atomic force microscope from Bruker (Santa Barbara, CA, USA) equipped with a Nanoscope V controller, which was operated in Peak Force (PF) Quantitative Nanomechanical Mapping (QNM) mode in air at room temperature. A scanning rate of about 1.0 Hz and a silicon tip with a resonance frequency of 75 kHz, a nominal radius of 10 nm, and a cantilever length of 225 µm were used for the measurements.

#### 2.4.4. XRD Analysis

The crystalline structures of celluloses before and after modifications were analyzed over the 2θ range from 10 to 40° using a SmartLab diffractometer (Rigaku, Japan) with Cu Kα radiation generated at 45 kV and 200 mA. The peaks corresponding to the different diffraction planes were deconvoluted using the Fityk 1.3.1. software [41], the interplanar distances were calculated using Bragg’s law, the crystal’s sizes were determined using the Scherrer equation, and the crystallinity degree was calculated by dividing the area of the crystalline peaks to the total area of the crystalline and amorphous peaks [42].

## 3. Results and Discussion

### 3.1. FTIR Analysis

**Figure 1:**
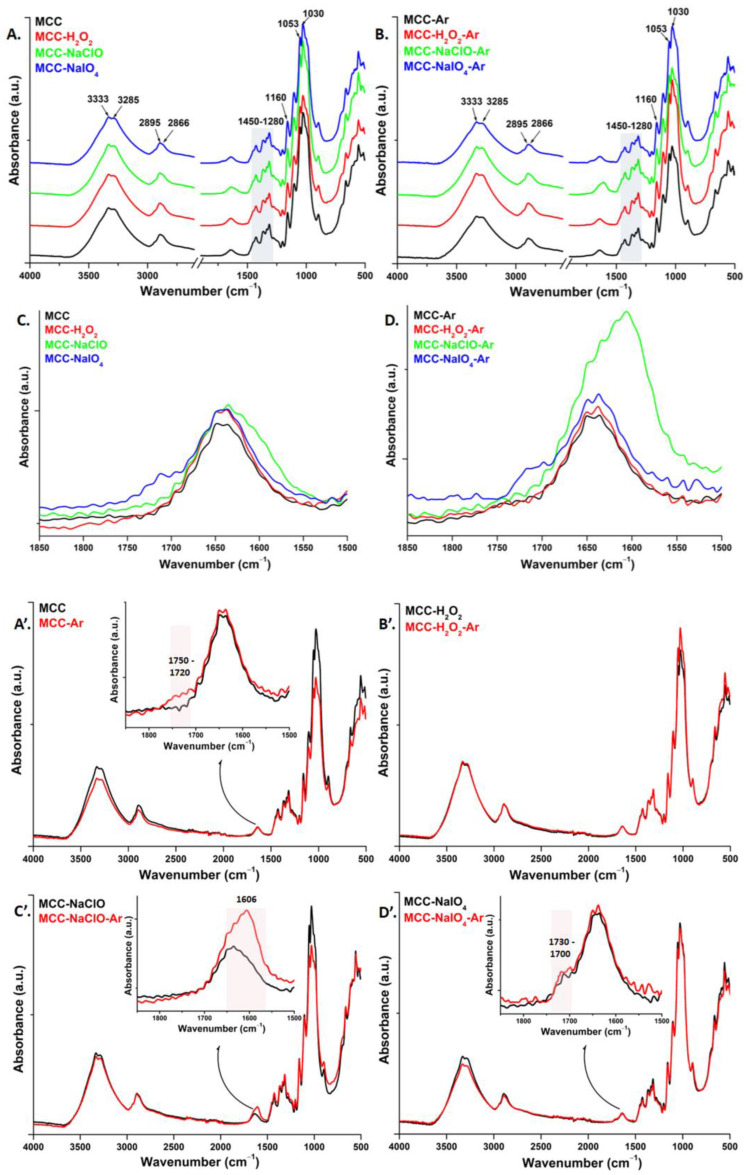
FTIR spectra of MCC (**A**) and plasma-treated MCC (**B**) in the presence of different oxidizing agents; detailed spectra (**C**,**D**); and comparative FTIR spectra of MCC (**A’**) and oxidized celluloses (**B’**–**D’**) before and after plasma treatment.

FTIR spectroscopy was carried out on the samples obtained by simply immersing MCC in water containing the oxidizing agents as well as on their counterparts that underwent plasma treatment in the presence of the oxidizing agents to assess the influence of the plasma treatment along with that of the oxidizing agents on cellulose surface chemistry. As it can be seen in Figure 1A, the FTIR spectrum of MCC shows peaks characteristic to the hydroxyl groups stretching at 3333 and 3285 cm^−1^, C–H stretching at 2895 and 2866 cm^−1^, C-H and O-H bending vibrations in the region 1450–1280 cm^−1^, and C-O-C and C-O stretching vibrations at 1160, 1053, and 1030 cm^−1^ [43,44,45]. All these characteristic bands of cellulose are also found in the FTIR spectra of MCC in contact with the oxidizing agents, with or without the plasma treatment (Figure 1A,B).

Regardless of the plasma treatment, the presence of H_2_O_2_ did not induce significant changes in the FTIR spectrum of cellulose. Indeed, the aldehyde groups that can be formed following plasma treatment in the presence of hydrogen peroxide are not stable and form hemiacetals or other compounds [46], whose infrared absorption bands overlap with those of cellulose. Moreover, the oxidation of cellulose using H_2_O_2_ is accompanied by the decomposition of cellulose and formation of soluble products [36], which were probably removed during the purification of the treated cellulose. However, several differences were observed between the FTIR spectrum of MCC and those of celluloses treated with the other two oxidizing agents. The FTIR spectrum of MCC-NaIO_4_ shows a small shoulder at 1715–1730 cm^−1^, which is much more obvious after the plasma treatment (Figure 1C,D). This shoulder corresponds to the C=O stretching vibration in aldehyde groups, which are formed following the periodate oxidation of cellulose [39,46]. In general, aldehyde groups are difficult to detect because they tend to stabilize themselves by forming hemiacetals, hydrates, and hemialdals [46]. Therefore, the shoulder observed at about 1730 cm^−1^ demonstrates the oxidization of the OH groups of MCC after treatment with sodium periodate and, especially, after treatment with sodium periodate in the presence of plasma.

Nevertheless, the most important modification in the FTIR spectra of celluloses was observed after the sodium hypochlorite treatment in the presence of plasma (Figure 1D). A new shoulder was observed for MCC-NaClO between 1560 and 1610 cm^−1^ in Figure 1C, while a large peak was observed in the FTIR spectrum of MCC-NaClO-Ar at ~1606 cm^−1^ (Figure 1D), showing the formation of the carboxylate groups on the surface of cellulose [35,47]. In addition, the large peak between 1570 and 1670 cm^−1^ in the FTIR spectrum of MCC-NaClO-Ar could also contain a contribution from the carbon–carbon double-bond group, indicating a more advanced degradation of cellulose under the influence of the plasma and NaClO oxidizing agent [48]. A further proof of the oxidation of the OH groups of cellulose was given by the decrease in the hydrogen bond intensity (HBI), which compares the absorption band at 3336 cm^−1^ with that at 1336 cm^−1^ and is related to the available hydroxyl groups for intramolecular hydrogen bonding [49,50]. The HBI decreased from 1.649 for MCC to 1.597 for MCC-NaClO, 1.467 for MCC-NaClO-Ar, 1.584 for MCC-NaIO_4_, and 1.571 for MCC-NaIO_4_-Ar, showing the conversion of the OH groups to a higher oxidation state [50]. Therefore, the FTIR results suggest a more intense effect of the oxidizing agents in the presence of plasma, especially in the case of NaClO treatment.

### 3.2. XPS Investigation

**Figure 2:**
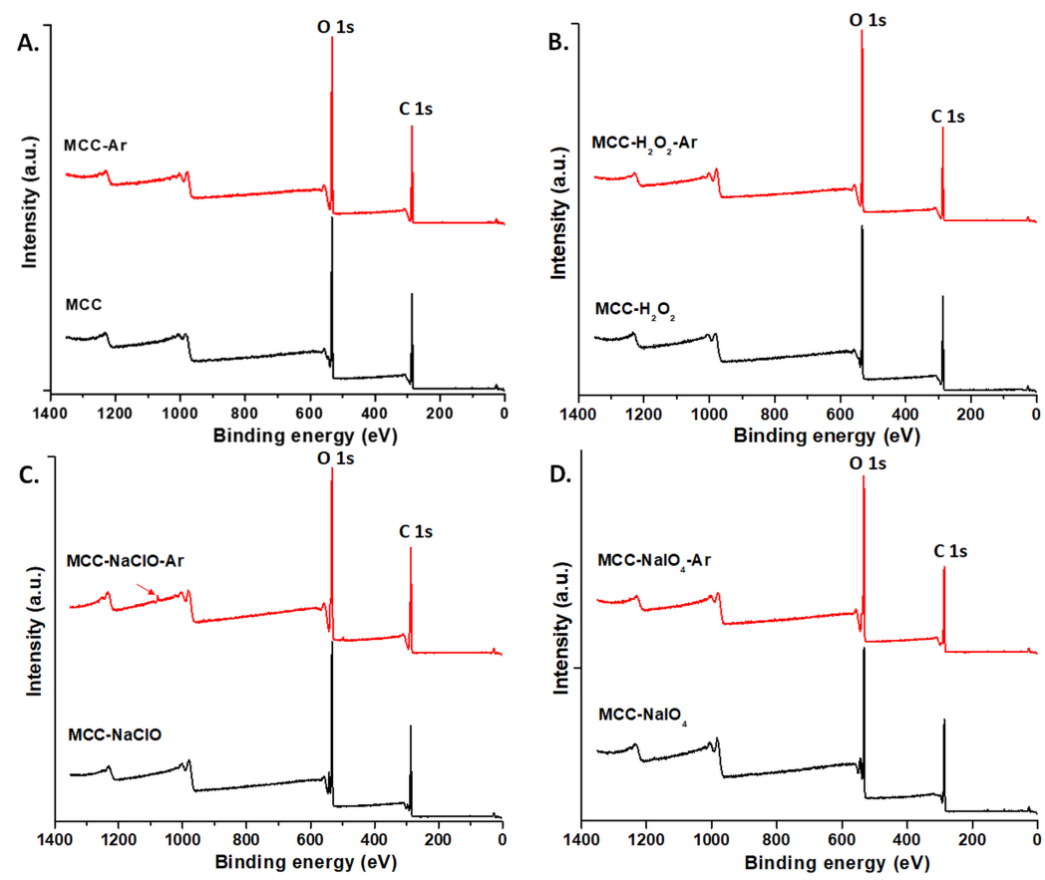
(**A**–**D**) XPS survey spectra of MCC treated with oxidizing agents with or without plasma.

The XPS survey spectra of the initial and differently treated cellulose samples are shown in Figure 2. The relative atomic percentages of carbon (C) and oxygen (O) are given in Table 1. Carbon and oxygen were the main constituents identified from the survey spectra (Figure 2) at the binding energies of 286.3 eV and 533.2 eV, respectively [35]. Due to the sample purification step, no contaminant elements were detected, except for a small peak at ~1075 eV. This small peak corresponds to the Na1s and confirms the presence of Na^1+^ traces in MCC-NaClO-Ar [51], probably bonded to the carboxylate anions that appeared in the FTIR spectrum (Figure 1D).

As observed in Table 1, Ar plasma treatment has oxidizing and cleaning effects in the absence of any oxidizing agent, leading to a slight increase in the O content and a corresponding decrease in the C content. In particular, the O/C ratio of MCC increased from 0.65 to 0.67 after the plasma treatment. The cleaning of the contaminants, usually characterized by a higher C content, from the surface of cellulose by the Ar plasma was also reported in previous works [31,32,52].

Plasma treatment in the presence of hydrogen peroxide led to an increase in the O/C ratio from 0.65 to 0.71, showing a clear oxidation effect, which was not detected in the absence of plasma. Indeed, hydrogen peroxide is a weak oxidizing agent in the absence of a metal catalyst or radiation, but it has been demonstrated that H_2_O_2_ decomposition is highly activated by plasma [53]. Therefore, it can be supposed that the action of plasma on hydrogen peroxide determined a significant increase in the concentration of highly reactive hydroxyl radicals (HO•), which oxidized MCC. Moreover, a previous study has shown that oxidation of cellulose using hydrogen peroxide and iron oxide catalysts is accompanied by cellulose depolymerization and formation of water-soluble products [47]; therefore, part of the oxidized cellulose could be lost during the purification step.

A strong increase in the O content was observed in the presence of sodium periodate, and this oxidizing effect was amplified when plasma treatment was applied to the MCC immersed in the periodate solution; the O/C ratio increased from 0.65 for unmodified MCC to 0.76 (periodate, no plasma) and, further, to 0.83 (periodate and plasma) (Table 1). It is known that the treatment of cellulose with sodium periodate is a chemo-selective reaction, which leads to the cleavage of the C2–C3 bond of the anhydroglucose unit and the formation of a dialdehyde [46]. Theoretically, the reaction proceeds with no change in the oxygen or carbon content and what changes is only the type of chemical bond [39,54]. However, previous works have shown that the newly formed dialdehyde cellulose undergoes cyclization, leading to hemiacetal and hemialdal structures in equilibrium with hydrated aldehyde forms [46,55], which are characterized by a larger O/C ratio. These trends explain the increased O/C ratio obtained after periodate oxidation of MCC and in the presence of plasma.

An increase in the O content was also observed when the MCC water suspension was mixed with sodium hypochlorite (Table 1). The increase in the O/C ratio is a common trend when cellulose is oxidized with different systems containing NaClO due to the formation of carboxyl groups instead of the hydroxyl groups bound to saturated carbon atoms in cellulose. In particular, the O/C ratio of a cellulose isolated from pineapple peel increased from 0.52 to 0.58 and 0.60 after the TEMPO oxidation with increasing amounts of NaClO (2.5 and 5 mmol, respectively, per 6 mmol of cellulose) [56]. Considering the greater amount of NaClO per cellulose that was used in our experiments, the higher increase in the O/C ratio was expected. A greater increase in the O/C ratio was also reported for cellulose fibers oxidized with NaClO in the presence of phthalimide-*N*-oxy radical, from 0.66 for the original cellulose to 0.75 for the oxidized cellulose [57]. However, a lower O/C ratio was obtained after the NaClO/plasma treatment (Table 1), suggesting that this treatment had a strong oxidative effect, with the formation of soluble oxidized products, as well as a destructive action, leading to small molecular weight products which, in general, are more soluble in water [12,58]. This hypothesis was verified by analyzing the oxidized cellulose immediately after the NaClO/plasma treatment and before the washing step. The XPS survey data showed, in this case, a very high O/C ratio of 1.47 (atomic % of O1s/C1s/Na1s/Cl2p = 43.0/29.3/13.0/14.7), showing the abundant formation of oxidized products with lower molecular weights.

**Figure 3:**
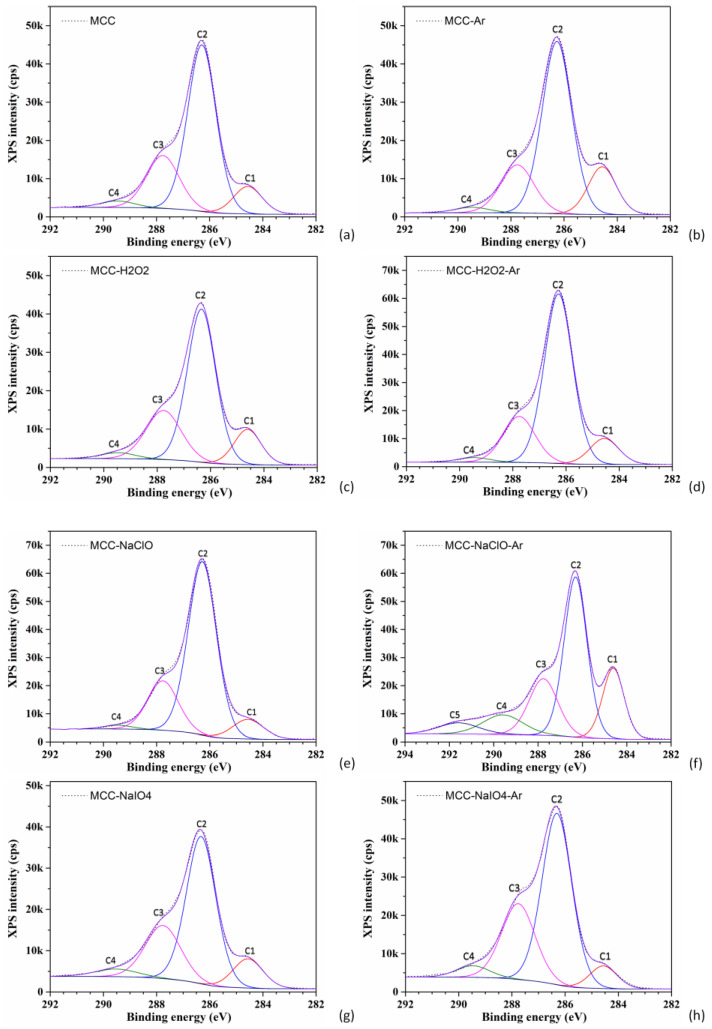
High resolution XPS spectra in the C1s regions with deconvolution: (**a**) MCC, (**b**) MCC-Ar, (**c**) MCC-H_2_O_2_, (**d**) MCC-H_2_O_2_-Ar, (**e**) MCC-NaClO, (**f**) MCC-NaClO-Ar, (**g**) MCC- NaIO_4_, and (**h**) MCC-NaIO_4_-Ar.

The high-resolution spectra from Figure 3 give more information about the chemical transformations that occur on the surface of the oxidized cellulose samples. The C1s region of MCC was fitted with three peaks: the first peak (C1), centered at a binding energy of 284.6 eV, was assigned to the C-C or C-H bonds from adventitious carbon; the second peak (C2), centered at 286.3 eV, was attributed to the C-O bonds in the C-OH groups and C-O-C linkages of cellulose; and the third peak (C3) at 287.9 eV was assigned to the O-C-O hemiacetal groups and to C=O bonds in oxidized species [31,32,59]. In addition to these main peaks, a small shoulder, which may be considered as a fourth peak (C4), was observed at about 289.5 eV. This new signal is, probably, determined by the presence of O=C-OH moieties. The relative atomic concentrations of C1s components in MCC, which are presented in Table 2, show that the contribution of this fourth component is under 3%.

For a deeper analysis, the ratio Cox/C2 was considered as an indicator of the oxidation degree [11]. It represents the proportion of oxidized components containing carbonyl and/or carboxyl groups (C3 and C4) related to the concentration of hydroxyl-bound carbon atoms (C2). Under the influence of plasma alone, hydrogen peroxide, or hydrogen peroxide coupled with plasma treatment, the concentration of C1s components in MCC varies slightly (Table 2), with a small decrease in the atomic concentration of oxidized species as compared to that of C2 being generally observed. This effect suggests the presence of impurities, with the cleaning effect of Ar plasma being known from previous works [31,32,60], similarly with that of hydrogen peroxide [61]. Another explanation for this result is the removal of water-soluble oxidized products during the purification step applied to the treated celluloses, a process that was reported in prior studies [11,47]. This effect could also explain the low Cox/C2 ratio in the case of the MCC-NaClO sample (Table 2). However, the oxidation of MCC with NaClO in the presence of plasma led to a strong increase in the concentration of oxidized components and the occurrence of a new peak at about 291 eV (Figure 3). This new peak (C5) may be ascribed to highly oxidized carbon components, such as carboxylates and carbonates [62,63]. The XPS results also showed a strong increase in the C1 component, which is ascribed to the carbon–carbon single and double bonds. Their presence may indicate advanced cellulose degradation; therefore, the cumulative effects of the NaClO oxidizing agent and plasma could also degrade MCC. Further, thermogravimetric analysis was used to verify this assumption. The presence of carboxylates and other degradation products on the surface of cellulose treated with NaClO and plasma was also signaled by FTIR. High oxidation levels (Cox/C2 ≥ 0.5) were also obtained in the presence of NaIO_4_, with or without plasma treatment (Table 2). The oxidation of MCC in the presence of periodate led to the formation of aldehyde groups, quantified in the XPS spectra by the increase in the C3 content, especially when plasma was also applied, and to the formation of higher oxidation products, which is signaled in the XPS spectra by the increase in the C4 contribution (Table 2). Therefore, the XPS data correlate well and complement the results obtained by FTIR.

### 3.3. Thermogravimetric Analysis

**Figure 4:**
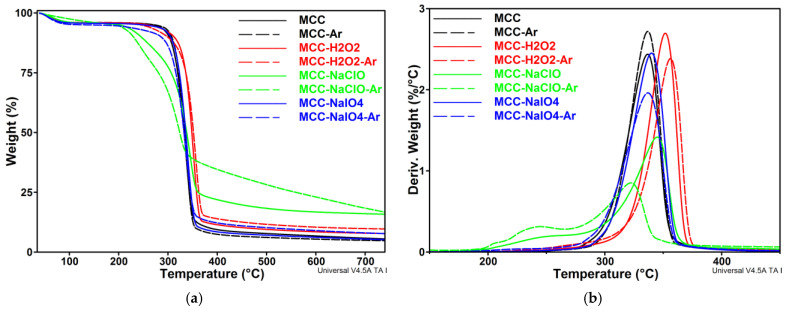
TG (**a**) and DTG (**b**) curves of differently treated celluloses.

Figure 4 shows the thermogravimetric (TG) and derivative (DTG) curves of MCC treated with different oxidizing agents in the presence or absence of plasma, while the most important thermal characteristics of the oxidized celluloses are listed in Table 3.

The effect of plasma on the thermal stability of cellulose samples is different in the absence of oxidizing agents than in their presence (Figure 4). Plasma treatment of MCC led to a slight increase in the thermal stability of MCC, which was illustrated by higher T_5%_ and T_onset_ values and lower R_700 °C_. These changes are, probably, determined by the cleaning effect of plasma. In the presence of oxidizing agents and depending on their nature, plasma treatment led to a lower thermal stability of the modified cellulose or to a slight increase in some characteristic temperatures (Table 3). In particular, MCC oxidized with hydrogen peroxide in the presence or absence of plasma showed a better thermal stability than the MCC reference, illustrated by higher T_onset_ and T_max_ values, while the T_5%_ value was lower, probably due to the presence of more volatile oxidized products. The increased thermal stability of MCC after the H_2_O_2_ treatment suggests a more ordered structure, probably determined by the selective action of hydrogen peroxide, which preferentially broke down the bonds in the amorphous regions of cellulose [36]. MCC oxidation with sodium periodate in the presence or absence of plasma brings small changes in the characteristic temperatures, except for the T_5%_ value, which is more than 100 °C lower than that of MCC for MCC-NaIO_4_-Ar. This obvious decrease in thermal stability at low temperatures is probably due to the presence of volatile oxidized products, such as low molecular weight dialdehyde and hemiacetal and hemialdal structures, as indicated by the FTIR and XPS analyses. Their presence is also indicated by the higher WL_100 °C_ value obtained in this case.

The TG and DTG curves of MCC-NaClO and MCC-NaClO-Ar have a different shape than those obtained for the other oxidized celluloses (Figure 4). A lower temperature shoulder or peak was noticed for these samples at around 250 °C, well before the temperature characteristic of cellulose degradation at the maximum rate (345 °C or 322 °C). The occurrence of the new peak shows the presence of carboxylated cellulose, characterized by a lower thermal stability [64], and other degradation products such as depolymerized oxidized cellulose. It has been reported that cellulose microfibers previously subjected to surface carboxylation by TEMPO-mediated oxidation using NaClO among other additives have shown two degradation steps, one at 240 °C, very close to the first degradation peak obtained for our samples, and the second one at 303 °C [65]. They explained the appearance of the first peak by decarboxylation of carboxyl groups of TEMPO-oxidized cellulose [65]. The high R_700 °C_ values obtained for NaClO-oxidized celluloses (Table 3) also suggest the presence of different degraded compounds compared to H_2_O_2_- or NaIO_4_-oxidized celluloses in the presence of plasma. These observations show that the results of thermogravimetric analysis correlate well with those obtained by FTIR and XPS and support the assumption that different, and, in some cases, cumulative mechanisms are involved in the oxidation/degradation processes of MCC using hydrogen peroxide, sodium hypochlorite, and sodium periodate agents.

### 3.4. XRD Results

**Figure 5:**
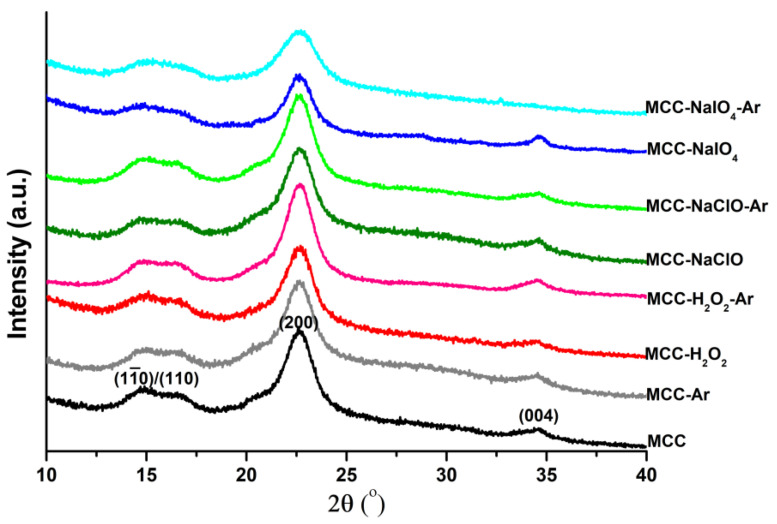
XRD patterns of differently treated celluloses; the diffraction planes are labeled according to Wada et al. (1993) [67].

In general, the amorphous phase of cellulose is more sensitive to oxidation or degradation reactions. Therefore, the crystallinity of MCC and the oxidized celluloses was determined by XRD to obtain more information about the changes induced by the oxidizing agents on the crystalline phase of cellulose. The XRD diagrams of cellulose samples (Figure 5) show the distinctive patterns of cellulose Iβ, which is characterized by a monoclinic unit cell, with the following 2θ (with θ being the Bragg’s angle) positions and corresponding lattice planes: 14.7° (1Ῑ0), 16.5° (110), 22.6° (200), 34.5° (004), and a shoulder at about 21° (102). These are similar to other observations [6,31,66,67]. The similar XRD patterns of the original MCC and treated celluloses support the idea that the treatment with oxidizing agents and/or plasma did not change the crystalline form of cellulose, but it induced some changes in the crystal structure. The most important parameters calculated from XRD data are disclosed in Table 4. Different effects of plasma, oxidizing agents, and plasma-oxidizing agents’ action are observed on the degree of crystallinity (X) of the cellulose samples. Plasma treatment led to a slight decrease in crystallinity. A similar effect was reported for MCC treated with plasma at atmospheric pressure using a plasma torch source for 30 min, when X decreased from 79.5% for MCC to 73.8% for the plasma-treated sample [68]. A very small variation in the X value, from 54.2% to 52.4%, was also reported for a cellulose nanocrystals’ film treated with a dielectric barrier discharge plasma in the presence of an Ar/silane gas mixture [69], while a strong decrease in X, from 78.2% (before treatment) to 60.5% (after treatment), was reported for a cellulose obtained from agave waste, which was exposed to a mechanical treatment by ball milling for 1 h [70]. It may be supposed that plasma led to a slight increase in the amorphous phase content due to its combined mechanical and chemical actions, which contribute to cellulose defibrillation, surface oxidation, and breaking hydrogen bonds. All these actions prevent, to a certain extent, the organization of the crystalline structures in cellulose [31,68].

The treatment of MCC with hydrogen peroxide, which is a weak oxidizing agent, did not change the X value, while NaIO_4_ and especially NaClO, which have a strong oxidizing effect, led to a clear decrease in crystallinity, with about 14% in the case of NaClO. This effect is due to the oxidative and degradation processes, which change the chemical structure of cellulose and decrease its molecular weight [71]. The concomitant action of oxidizing agents and plasma had different effects on the crystallinity of cellulose, with MCC-H_2_O_2_-Ar showing a higher X value, while MCC-NaClO-Ar and MCC-NaIO_4_-Ar had lower X values (Table 4). It can be supposed that, in the first case, the oxidizing effect of hydrogen peroxide and the cleaning/oxidizing effect of plasma are not accompanied by degradative changes in MCC, while, in the latter cases, the oxidative and degradative processes take place simultaneously. These results correlate well with the FTIR, XPS, and TGA results, which showed the slight oxidative and cleaning effects of hydrogen peroxide and plasma in contrast to the strong oxidative and degradative effects of the other two oxidizing agents.

The interplanar spacing (d) and crystal size (D) were also measured perpendicular to the (200) plane because the (200) peak was the most clearly resolved (Figure 5). As shown in Table 4, regardless of the treatment applied, no significant variations in the d values were observed, only small variations in the crystal size being recorded after treatments. After analyzing the XRD data, it can be concluded that a clear consumption of the amorphous phase in the oxidative processes, having as a result an increase in crystallinity, was observed only for the H_2_O_2_/plasma-treated MCC. It may be presumed that the hydroxyl radicals, generated following the decomposition of hydrogen peroxide, have attacked the less organized regions on the surface of cellulose microfibrils, leading to lower molecular weight oxidized species, which dispersed easily in water, thus increasing the content of the crystalline regions, which were more resistant to the oxidative attack [36]. The treatment of MCC with the other two oxidizing agents in the presence of plasma led to strong oxidative and degradative processes, which probably started by attacking the amorphous phase but over time damaged both the amorphous and crystalline phases, causing a decrease in the degree of crystallinity.

### 3.5. Morphological Investigation

#### 3.5.1. SEM Analysis

**Figure 6:**
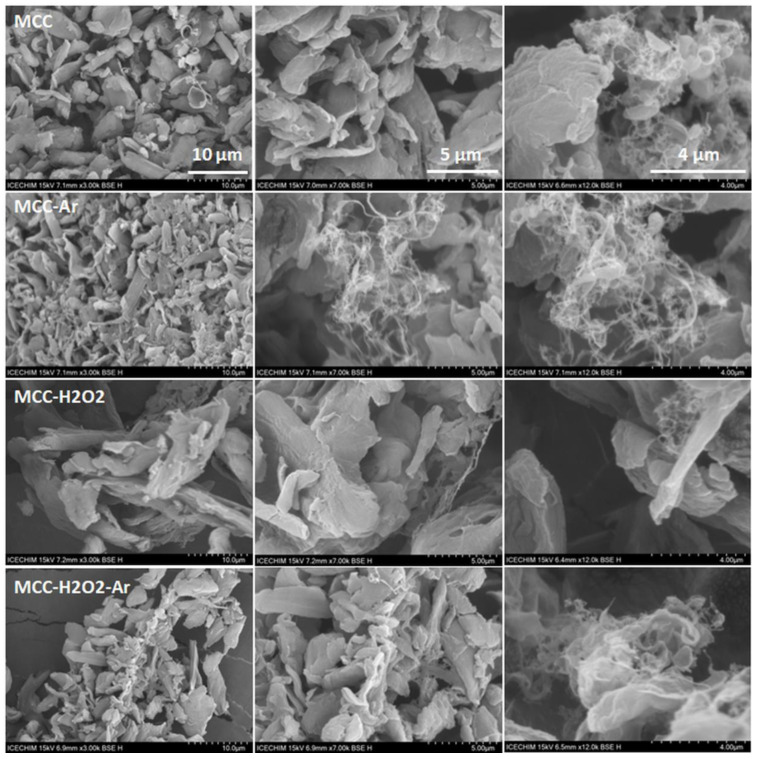
SEM images of MCC, MCC-Ar, MCC-H_2_O_2_, and MCC-H_2_O_2_-Ar, with magnifications of 3000×, 7000×, and 12,000×, from left to right.

The SEM analysis provides us with important information regarding the changes that occur in the morphology and size of the MCC particles following their treatment with different oxidizing agents, in the presence or absence of plasma. This is important as it allows us to assess the degree of fragmentation and defibrillation that each of the explored treatments induces on cellulose. As shown in the SEM images (Figure 6), untreated MCC consists of irregular-shaped particles [72], with a low aspect ratio [31] and a pronounced tendency of agglomeration [73]. At higher magnification (12,000×), regions where thinner cellulose fibers form a loose, lace-like network can be observed. These can be attributed to the partial defibrillation of MCC during the ultrasonication stage used in this work for the preparation of the aqueous dispersion of MCC. Ar plasma treatment induced a partial fragmentation of the MCC, visible by the breakdown of the MCC particles into smaller structures. The higher magnification images revealed that, in certain areas, the plasma treatment induced the defibrillation of MCC, evidenced by the splitting of the particles into thinner fibers and the detachment from the bulk structures of thin fibers [31] that form a loosely packed, porous network.

Treatment with H_2_O_2_ alone did not bring noticeable changes in the morphology or dimensions of the MCC particles (Figure 6). This was to be expected considering that H_2_O_2_ is a weak oxidizing agent [74], and no catalyst was used for the activation of H_2_O_2_ decomposition [75]. Indeed, the treatment of cellulose with H_2_O_2_ in the presence of trace amounts of CuSO_4_·5H_2_O as a catalyst, at 60 °C for 72 h and in an acidic medium (pH 1–2) led to the fragmentation of cellulose into nanocrystals [76]. The SEM images from Figure 6 also show that, when plasma-assisted, H_2_O_2_ induced significant erosion and fragmentation of the MCC particles, transforming the once-compact MCC particles into smaller, disintegrated structures, characterized by a higher degree of order. According to the literature, Ar plasma can activate the decomposition of H_2_O_2_ [77] into highly reactive species, such as hydroxyl radicals (HO•) that can oxidize or break bonds in cellulose [76]. From the obtained micrographs, plasma-activated H_2_O_2_ appears to have acted as a selective degradation agent that broke down certain regions in the MCC particles, while leaving others virtually intact. Given their higher accessibility and susceptibility to chemical attack, it can be presumed that the plasma-activated H_2_O_2_ preferentially degraded the amorphous regions from the MCC particles, while preserving their crystalline domains. This presumption is in agreement with the XRD results that showed a significant increase in the crystallinity degree of MCC after the combined Ar plasma/H_2_O_2_ treatment. The higher magnification images (12,000×) are indicators for the defibrillating effect of the combined Ar plasma/H_2_O_2_ treatment, showcasing the disruption of the original compact MCC structures with the formation of a more open structure consisting of thin fibers and sheets assembled into a loosely packed, porous network. Indeed, plasma-assisted H_2_O_2_ treatment can oxidize the OH groups or break the glycosidic bonds in cellulose, thereby weakening the microfibril network structure and facilitating the defibrillation [78].

**Figure 7:**
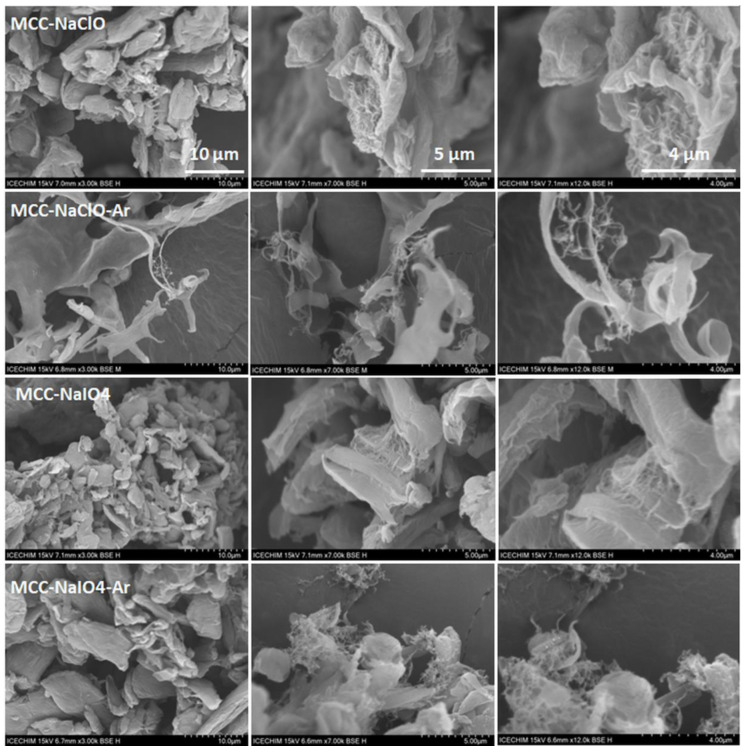
SEM images of MCC-NaClO, MCC-NaClO-Ar, MCC-NaIO_4_, and MCC-NaIO_4_-Ar, with magnifications of 3000×, 7000×, and 12,000×, from left to right.

Unlike H_2_O_2_, NaClO is a strong oxidizing agent [79] that led to a certain degree of fragmentation of MCC even in the absence of Ar plasma (Figure 7). As compared to the untreated MCC, the surfaces and edges of the MCC-NaClO particles displayed increased roughness due to the chemical degradation and surface etching caused by the oxidative action of NaClO. Higher magnification (7000× and 12,000×) images revealed that NaClO degraded MCC, exhibiting an exfoliating or peeling-like effect. The exposed, exfoliated areas consist of highly disrupted fibrillar structures assembled into a loose, porous network.

In conjunction with Ar plasma, the disintegrating action of NaClO is intensified, the MCC particles being transformed almost completely into very thin sheet-like or folded ribbon-like structures, while highly disrupted fibrils that form loosely packed, porous networks were also noticed in multiple regions (Figure 7). It is possible that the combined treatment with Ar plasma and NaClO caused an advanced defibrillation of MCC, yielding predominantly nanofibers which, during freeze-drying, self-assembled into the observed film- and ribbon-like structures. This phenomenon of lateral self-assembly of nanofibers into bulk morphologies (wide fibers, ribbons, films, etc.) was also observed for a 0.05 wt% aqueous suspension of TEMPO-oxidized CNFs following rapid liquid nitrogen freezing and freeze-drying [80]. The more advanced defibrillation of MCC following the plasma assisted-NaClO treatment as compared to other oxidizing agents is to be expected, given that a substantially higher level of oxidation was revealed by the XPS analysis in this case. The plasma-assisted oxidation with NaClO likely converted the C-OH groups from cellulose into aldehyde or carboxylate groups, which led to a decrease in the number of hydrogen bonding interactions and/or an increase in the electrostatic repulsions between the cellulose chains, facilitating defibrillation [81].

Treatment with NaIO_4_ produced advanced fragmentation of the MCC particles, visible by their breakdown into smaller fragments (Figure 7). Increased magnifications (7000× and 12,000×) revealed that MCC-NaIO_4_ particles exhibited layered and roughened surfaces, which is an indicator of cellulose degradation, as well as partial defibrillation, noticeable by the detachment of fine fibers from the bulk material. As with the other two oxidizing agents, Ar plasma augmented the disintegrating action of NaIO_4_, causing, as revealed by the high magnification (7000× and 12,000×) micrographs, an advanced defibrillation of the MCC into very thin fibers. The sheet- or ribbon-like structures observed from place to place can result from the association of adjacent fibrils following freeze-drying. During freezing, the growth of ice crystals or sheets can push cellulose fibrils closer. Subsequently, removal of ice during sublimation leaves behind a structure in which the fibrils, having aggregated, become inter-connected by H-bonding interactions and entanglements [82] and organize into large ribbon- or sheet-like formations. Similar to the NaClO case, the combined NaIO_4_/plasma treatment of cellulose leads to the conversion of some of the C-OH into aldehyde groups, disrupting hydrogen bonds in cellulose [83] and promoting defibrillation.

In all instances, Ar plasma treatment enhances the degrading and defibrillating actions of the chemical oxidizing agents. This may be due to the plasma treatment’s ability to generate highly reactive species that can enhance or complement the oxidative and degradative action of the chemical oxidizing reagents, as well as due to its capacity to disrupt the hydrogen bonding interactions in cellulose [6], ensuring a better accessibility of the oxidizing agents to the cellulose structure.

#### 3.5.2. AFM Investigation

**Figure 8:**
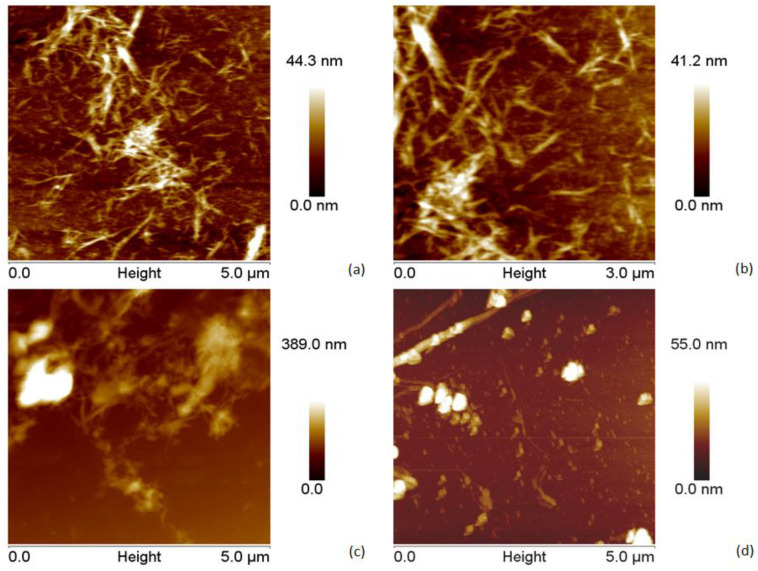
AFM images of MCC-NaClO-Ar (**a**,**b**), MCC-NaIO_4_-Ar (**c**), and MCC-H_2_O_2_-Ar (**d**).

The different effects of the oxidizing agents in the presence of plasma on MCC’s morphology was also highlighted by the AFM images (Figure 8).

The NaClO/plasma treatment led to an advanced fragmentation and defibrillation of MCC, with the formation of a network of cellulose nanofibers over large areas (Figure 8a,b). An average width of 32.2 ± 14.5 nm was calculated for the cellulose nanofibers produced by the NaClO/plasma treatment. The NaIO_4_/plasma treatment led to the fragmentation of MCC particles in smaller and thinner fragments and, also, to a certain degree of defibrillation (Figure 8c). Cellulose nanofibers with a width of 53.3 ± 18.5 nm, which are interspersed with coarser particles of 200–400 nm and agglomerations of cellulose nanofibers, can be observed in Figure 8c as a result of the NaIO_4_/plasma treatment of MCC. Similar to the SEM results, the AFM images of MCC treated with hydrogen peroxide and plasma revealed the advanced fragmentation of the MCC particles into smaller ones, but only rarely the presence of cellulose nanofibers as an effect of defibrillation.

In summary, the morphological analysis of MCC treated with oxidizing agents and plasma highlights the differentiated role of chemical and plasma treatment in cellulose fragmentation and defibrillation, with the most intense defibrillation effect and the obtaining of a large proportion of nanofibers occurring with the NaClO/plasma treatment.

## 4. Conclusions

This study proposes a simple and efficient method to simultaneously defibrillate and oxidize cellulose using submerged Ar plasma treatment of aqueous MCC dispersions that also contain a chemical oxidizing agent, such as H_2_O_2_, NaClO, or NaIO_4_. The present study highlights, for the first time, the effect of plasma to enhance the action of various oxidizing agents on cellulose. The plasma-assisted treatment with oxidizing agents in solution emerges as a clean strategy to enhance the oxidation of cellulose or shorten the reaction time. The advantage of plasma-assisted oxidation of cellulose was highlighted by structural analyses, with the FTIR and XPS results showing combined cleaning and oxidative actions of the treatment, and, in the case of NaClO, also a controllable destructive action, which was confirmed by TGA. The XRD analysis showed that the H_2_O_2_/plasma treatment increased the crystallinity of MCC, while NaClO/plasma and NaIO_4_/plasma treatments decreased it, indicating that H_2_O_2_ preferentially degraded the amorphous regions in MCC, while NaClO and NaIO_4_ were more aggressive, also attacking the crystalline domains in MCC. More significantly, the combined treatments with plasma and chemical oxidants affected the morphology and dimensions of the MCC particles, leading to the fragmentation of MCC and to varying degrees of defibrillation, as illustrated by the SEM and AFM images. Regarding future perspectives, this study deserves to be deepened by varying different parameters to optimize the process for targeted applications.

The obtained oxidized cellulose has a plethora of potential applications in bio-medicine (as a hemostatic agent, antimicrobial and absorbent material, scaffold for tissue regeneration, drug delivery system, etc.), in packaging, in water depollution, or as a stabilizer or thickener in the food, pharmaceutical, and cosmetic industries. Additionally, due to the new functional groups generated by oxidation, oxidized cellulose can serve as a promising platform for further chemical modifications, which can vastly expand its applications.

## Figures and Tables

**Table 1:** The relative atomic percentages of C and O in the MCC and modified cellulose samples.

Atomic Concentration, %	MCC	MCC-Ar	MCC-H_2_O_2_	MCC-H_2_O_2_-Ar	MCC-NaClO	MCC-NaClO-Ar	MCC-NaIO_4_	MCC-NaIO_4_-Ar
C1s	60.8	59.9	61.4	58.5	54.1	61.5	56.9	54.7
O1s	39.2	40.1	38.6	41.5	45.9	38.5	43.1	45.3
O/C ratio	0.65	0.67	0.63	0.71	0.85	0.63	0.76	0.83

**Table 2:** The relative atomic concentrations of differently bound carbon atoms in the oxidized cellulose samples.

C1s Components	MCC	MCC-Ar	MCC-H_2_O_2_	MCC-H_2_O_2_-Ar	MCC-NaClO	MCC-NaClO-Ar	MCC-NaIO_4_	MCC-NaIO_4_-Ar
C1 (%)	10.6	15.4	13.1	10.8	8.4	18.8	11.8	7.1
C2 (%)	63.3	62.8	60.2	64.9	68.9	45.6	58.6	59
C3 (%)	23.2	19.5	23.8	21.9	21.2	20.3	25.4	29.5
C4 (%)	2.9	2.3	2.9	2.4	1.5	9.7	4.2	4.4
C5 (%)	-	-	-	-	-	5.6	-	-
Cox/C2	0.41	0.35	0.44	0.38	0.33	0.78	0.50	0.57

**Table 3:** Thermogravimetric characterization results for MCC treated with different oxidizing agents and plasma (WL_100°C_—weight loss at 100 °C, T_5%_—temperature at 5% weight loss, T_onset_—onset degradation temperature, T_max_—temperature at the maximum degradation rate, and R_700°C_—residue at 700 °C).

Thermal Characteristics	MCC	MCC-Ar	MCC-H_2_O_2_	MCC-H_2_O_2_-Ar	MCC-NaClO	MCC-NaClO-Ar	MCC-NaIO_4_	MCC-NaIO_4_-Ar
WL_100 °C_ (%)	4.0	3.9	3.9	3.9	3.9	3.3	4.2	4.8
T_5%_ (°C)	268.9	273.2	259.4	245.3	202.6	192.5	250.1	146.4
T_onset_ (°C)	314.2	317.3	332.2	334.3	311.0	275.0	318.1	312.0
T_max_ (°C)	336.7	336.9	351.8	356.3	344.9(250)	322.3(245)	340.0	336.9
R_700 °C_ (%)	5.8	4.9	7.9	9.8	16.1	18.4	5.9	8.0

**Table 4:** XRD parameters obtained after deconvolution: degree of crystallinity (X), d-spacing (d), and crystallite size perpendicular to the (200) plane (D).

XRD Parameters	MCC	MCC-Ar	MCC-H_2_O_2_	MCC-H_2_O_2_-Ar	MCC-NaClO	MCC-NaClO-Ar	MCC-NaIO_4_	MCC-NaIO_4_-Ar
X, %	67.7	64.9	67.3	73.2	58.2	62.5	64.1	60.7
d, nm	0.392	0.393	0.392	0.392	0.392	0.393	0.391	0.391
D, nm	4.9	4.6	5.0	5.1	4.7	4.8	5.1	4.6

## Data Availability

The original contributions presented in this study are included in the article. Further inquiries can be directed to the corresponding authors.
